# Broadband Prosthetic Interfaces: Combining Nerve Transfers and Implantable Multichannel EMG Technology to Decode Spinal Motor Neuron Activity

**DOI:** 10.3389/fnins.2017.00421

**Published:** 2017-07-19

**Authors:** Konstantin D. Bergmeister, Ivan Vujaklija, Silvia Muceli, Agnes Sturma, Laura A. Hruby, Cosima Prahm, Otto Riedl, Stefan Salminger, Krisztina Manzano-Szalai, Martin Aman, Michael-Friedrich Russold, Christian Hofer, Jose Principe, Dario Farina, Oskar C. Aszmann

**Affiliations:** ^1^CD-Laboratory for the Restoration of Extremity Function, Department of Surgery, Medical University of Vienna Vienna, Austria; ^2^Department of Hand, Plastic and Reconstructive Surgery, Burn Center, BG Trauma Center Ludwigshafen, Plastic and Hand Surgery, University of Heidelberg Ludwigshafen, Germany; ^3^Department of Bioengineering, Centre for Neurotechnology, Imperial College London London, United Kingdom; ^4^Neurorehabilitation Systems Research Group, Clinic for Trauma Surgery, Orthopedics and Plastic Surgery, University Medical Center Göttingen Göttingen, Germany; ^5^Health Assisting Engineering, University of Applied Sciences Wien Vienna, Austria; ^6^Division of Plastic and Reconstructive Surgery, Department of Surgery, Medical University of Vienna Vienna, Austria; ^7^Otto Bock Healthcare Products GmbH Vienna, Austria; ^8^Department of Electrical and Computer Engineering, University of Florida Gainesville, FL, United States

**Keywords:** myoelectric prosthesis, prosthetic interface, EMG, nerve transfers, TMR, targeted muscle reinnervation, prosthetic control

## Abstract

Modern robotic hands/upper limbs may replace multiple degrees of freedom of extremity function. However, their intuitive use requires a high number of control signals, which current man-machine interfaces do not provide. Here, we discuss a broadband control interface that combines targeted muscle reinnervation, implantable multichannel electromyographic sensors, and advanced decoding to address the increasing capabilities of modern robotic limbs. With targeted muscle reinnervation, nerves that have lost their targets due to an amputation are surgically transferred to residual stump muscles to increase the number of intuitive prosthetic control signals. This surgery re-establishes a nerve-muscle connection that is used for sensing nerve activity with myoelectric interfaces. Moreover, the nerve transfer determines neurophysiological effects, such as muscular hyper-reinnervation and cortical reafferentation that can be exploited by the myoelectric interface. Modern implantable multichannel EMG sensors provide signals from which it is possible to disentangle the behavior of single motor neurons. Recent studies have shown that the neural drive to muscles can be decoded from these signals and thereby the user's intention can be reliably estimated. By combining these concepts in chronic implants and embedded electronics, we believe that it is in principle possible to establish a broadband man-machine interface, with specific applications in prosthesis control. This perspective illustrates this concept, based on combining advanced surgical techniques with recording hardware and processing algorithms. Here we describe the scientific evidence for this concept, current state of investigations, challenges, and alternative approaches to improve current prosthetic interfaces.

## Introduction

Upper extremity loss is a severe lifetime event leading to a significant physical and consecutive psychological burden to patients. In modern extremity reconstruction, myoelectric prostheses are used to restore limb function (Kung et al., 2013; Farina and Aszmann, 2014). These devices rely on detection of voluntary residual muscle activity through electromyography (EMG) to drive prosthetic function. However, current prosthetic interfaces are unable to provide sufficient, intuitive and reliable control, which is one of the main reasons for device abandonment (Atkins et al., 1996; Biddiss et al., 2007; Peerdeman et al., 2011). Novel control interfaces are therefore needed to provide a robust broadband link between the patient and the prosthesis (Peerdeman et al., 2011; Ortiz-Catalan et al., 2012; Kung et al., 2013).

Since the 1960s, the prosthetic interface has utilized the amputee's two major muscle groups of the residual stump as sources of myocontrol signals (Childress, 1985; Williams, 1990; Parker et al., 2006). This approach is simple, reliable, and non-invasive, but the information transfer is limited since only two control signals from an agonist-antagonist muscle pair are available. In this scenario, co-contraction is often used to switch the control signals to different joints of the prosthetic device (hand, wrist, elbow or shoulder) (Salminger et al., 2015; Vujaklija et al., 2016). This classic control method is unintuitive and cumbersome but, due to its reliability, it is still the only widely applied clinical solution (Turker, 1993; Scheme and Englehart, 2011; Farina and Aszmann, 2014). Considering their complexity, it is apparent that the modern prostheses cannot be controlled intuitively with this traditional interface (Ortiz-Catalan et al., 2012; Kung et al., 2013).

**Figure 1:**
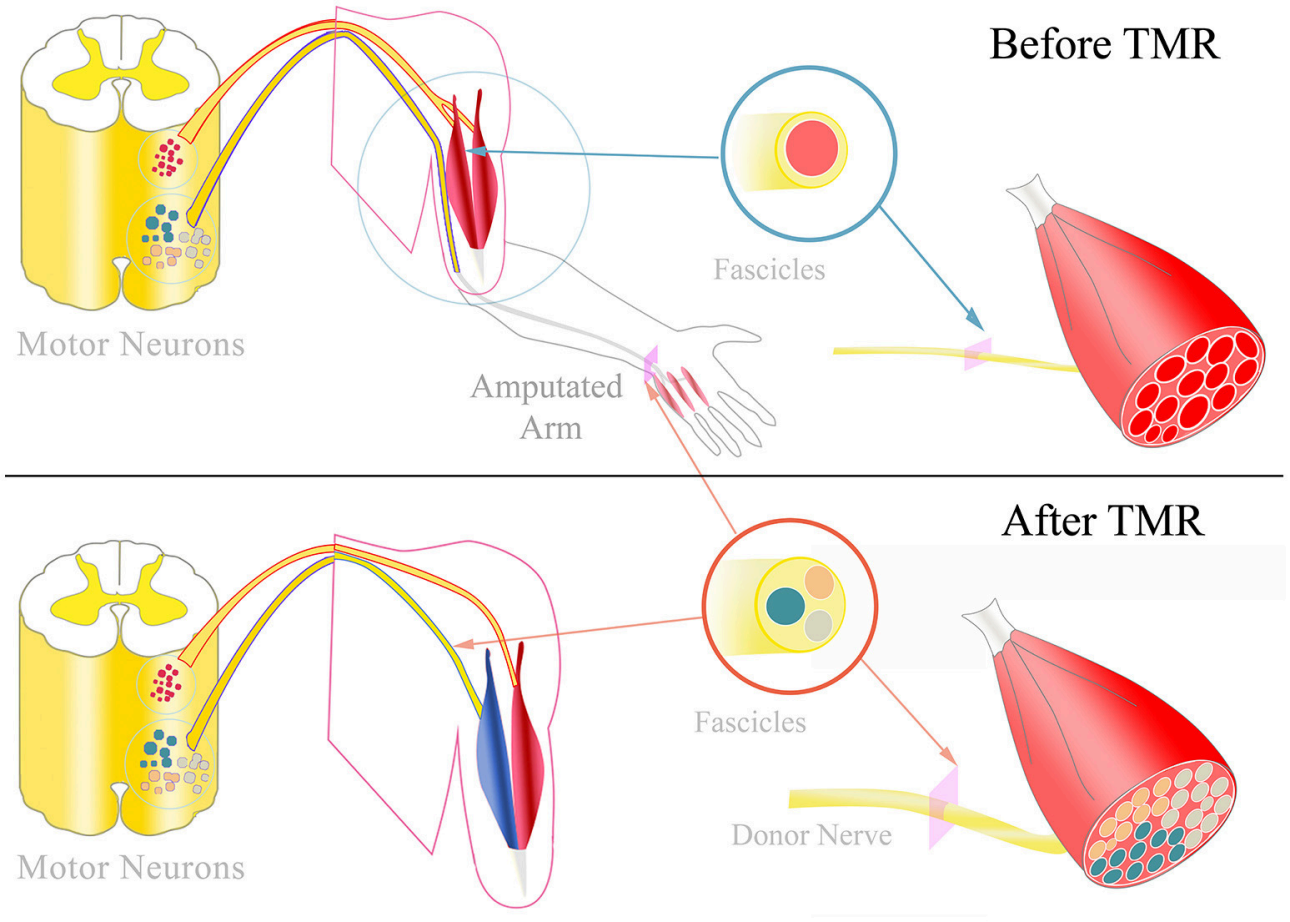
TMR and hyper-reinnervation: **Top:** Physiologically, peripheral nerves typically innervate multiple muscles via different motor fascicles. The fascicles' motor neurons are located in the motor neuron columns of the spinal cord. Each motor neuron innervates a certain number of muscle fibers, termed the muscle unit. After amputation, these motor neurons and fascicles remain intact without any function. **Bottom:** During TMR surgery, amputated nerves are transferred to replace the target muscle's original motor nerve. The donor nerve is typically a multi-fascicular nerve that includes a higher number of motor neurons. Consequently, the targeted muscle is hyper-reinnervated by more motor neurons which form smaller muscle units. Additionally, the individual motor fascicles could form fascicular territories within the muscle that could potentially contract independently from each other.

Targeted muscle reinnervation (TMR) was proposed to increase the number of intuitive control signals in prosthetics (Kuiken et al., 2005; Miller et al., 2008). With this approach, peripheral nerves deprived of functional targets due to amputation are transferred to residual muscles in the stump and the original motor branch divided (Kuiken et al., 2009; Kung et al., 2013; Figure 1). As a consequence, the innervation of multi-headed or segmentally innervated muscles (e.g., biceps, pectoralis muscle) is separated to increase the number of available myosignals (Dumanian et al., 2009). TMR thus enables simultaneous control of multiple degrees of freedom (DoFs), such as hand opening, wrist rotation and elbow flexion (Kuiken et al., 2007; Salminger et al., 2015). In addition, TMR signals are intuitive to use for the patient, as the nerve's original function is the same function as controlled in the prosthesis after the surgery. However, the latest prosthetic devices allow in principle highly dexterous hand-like motions involving multiple DoFs, including individual finger motions (Fifer et al., 2014; Lee et al., 2014; Resnik et al., 2014). For controlling these functions, an even greater number of control signals than TMR currently provides are needed.

Previous investigations indicate that TMR leads to hyper-reinnervation and cortical reafferentiation (Kuiken et al., 1995). By recording the activity of the reinnervated muscles with multichannel EMG systems, activation patterns of single motor units can potentially be decoded. Based on these patterns, we believe it is possible to estimate the neural drive for complex tasks from the spinal cord. This could provide a broadband interface for the user's motion intention and thus govern modern myoelectric prostheses in a natural manner. In this perspective article, we present these concepts and the scientific foundation for their clinical translation.

## The neurophysiology of targeted muscle reinnervation

During TMR surgery, nerves that have lost their target due to amputation are transferred to residual stump muscles to increase the number of cognitive and independent muscle signals. In this procedure, the original motor branch of a redundant muscle is replaced by an amputated nerve and thus this muscle is reinnervated by a different pool of motor neurons that previously encoded hand function (Figure 1). Consequently, the target muscle function is controlled by a different segment of the spinal cord and cortex area with respect to its natural innervation. Given sufficient recovery time, TMR leads to the representation of the targeted muscle at the original cortical location of the missing limb (Chen et al., 2013; Yao et al., 2015). This reafferentiation of the highly adapted corticospinal control structures of the lost extremity creates intuitive signals for prosthetic use. In this process, the corticospinal areas originally linked to the fine motions of the hand are reconnected to proximal muscles.

Essential for cognitively establishing such a high number of control signals is a structured feedback-driven neurorehabilitation program. For this purpose, EMG biofeedback is used to facilitate motor learning and to teach patients how to activate the newly established muscle signals. Following surgery, cortical plasticity allows the patient to reintegrate the rewired neuromuscular structures and use them to intuitively control a prosthesis in daily activities (Stubblefield et al., 2009; Sturma et al., 2014).

### Motor unit structure

TMR alters the components of the motor units and may thereby change their physiology. During maturation of the nervous system, the motor neuron, connecting axon, neuromuscular junction and muscle fibers are physiologically aligned in their properties (Buchthal and Schmalbruch, 1980; Heckman and Enoka, 2012). During the nerve transfers used in TMR, motor neurons and their axons are linked to new muscle fibers with potentially different properties than the original fibers. As cross-innervation studies have shown (Prewitt and Salafsky, 1967; Romanul and Van Der Meulen, 1967; Amphlett et al., 1975), the reinnervating motor neurons may restore the integrity of the motor unit by changing the myosin heavy chain expression of the muscle fibers. Although the extent of this transition requires further investigation in TMR, this mechanism transforms the targeted muscle fibers into fibers with similar characteristics as in the originally innervated muscle. Hence, the amputee would be equipped with a complex polytopic muscle signal that is physiologically similar to that of the amputated musculature and could provide a “bioscreen” of the EMG activity of lost natural muscles. This activity can then be mapped into natural control signals.

### Hyper-reinnervation

Nerve transfer with an axonal surplus can lead to hyper-reinnervation of the targeted muscle (Kuiken et al., 1995). This reinnervation by greater motor neuron numbers leads to an increased number of smaller functional motor units (Kuiken et al., 1995; Kapelner et al., 2016), so that targeted muscles can potentially be controlled in a finer way than with their original innervation. This property can be exploited for optimal control with a precise tuning between the number of reinnervating axons and the available target muscle fibers to reach an optimal level of hyper-reinnervation. Additional axonal surplus could be diverted to a different target muscle to further increase the overall number of control sites and/or generate redundancy for increasing the robustness of the control system.

### Fascicular territories within the targeted muscle

Following TMR, donor nerves originally innervating multiple muscles with similar functions (e.g., ulnar nerve innervating intrinsic muscles of the hand) are transferred to only one target muscle (Aszmann et al., 2008). Thus, individual nerve fascicles may innervate different portions of the target muscle, corresponding to the muscles originally innervated by, e.g., the ulnar nerve. These targeted muscle portions may be in principle independently controlled (Figure 1). Therefore, the original innervation capacity of the nerve could be projected within one muscle, with the limitations of the available muscle fibers and of the neurotrophic support for only a part of the donor nerve (Kuiken et al., 1995). Classic EMG recordings currently used for prosthesis control cannot discriminate between the activities of clusters of muscle fibers from the same muscle. Nonetheless, more selective EMG techniques, as for example implantable electrodes, may allow such discrimination, so that multi-fascicular nerves could be interfaced from recordings from a single muscle. Consequently, EMG signal recording and decoding systems that allow the identification of the activity of motor units controlled by different nerve fascicles within the same muscle could extract the original neural code for fine motor control. Fascicular territories within the targeted muscle could be accessed with highly selective implanted electrodes to record a higher number of individual control signals.

## Multichannel EMG systems

Muscle signals are spatially separated during TMR surgery to allow recording with standard surface electrodes (Dumanian et al., 2009). The resulting EMG signals are largely independent and each able to reliably control one prosthetic function. An increase in number of recording sites would provide more EMG signal sources but would increase their correlation due to volume conduction.

Modern implantable EMG systems can acquire EMG signals in close proximity to the muscle fibers. Moreover, implantable systems ensure stable relations between the sources and the electrodes. In contrast, surface EMG records from a larger muscle volume and is subject to external factors, such as sweat or displacement. Recent investigations of chronic implantable systems, such as the MyoPlant system (Lewis et al., 2013b) and the IMES (Troyk et al., 2007; Merrill et al., 2011), have shown that they can provide superior EMG data quality than surface recordings. These systems are implanted into the patient's extremity for recording and wireless transmission of high-quality muscle signals. EMG data are recorded either from within the muscle (e.g., IMES) or epimysially from the muscle surface (e.g., MyoPlant). Deep and/or small muscles that cannot be easily monitored with non-invasive EMG electrodes can be targeted by muscle implants, resulting in an increase in control capabilities. The performance of the IMES system has been tested in a first clinical trial with implantation of eight electrodes in transradial amputees showing safe application and simple yet efficient control over multiple DoFs (Weir et al., 2003; Baker et al., 2010; Pasquina et al., 2014). The MyoPlant system has been successfully tested in extensive pre-clinical large animal trials, showing good biocompatibility and stable electrode impedance over the course of several months, and reliable acquisition of multiple adjacent EMG signals (Poppendieck et al., 2011; Lewis et al., 2013a; Bergmeister K. D. et al., 2016). In view of a chronic application, further investigations are needed for longer-term periods. These chronic studies are currently being conducted for the IMES system by our and several other research groups.

**Figure 2:**
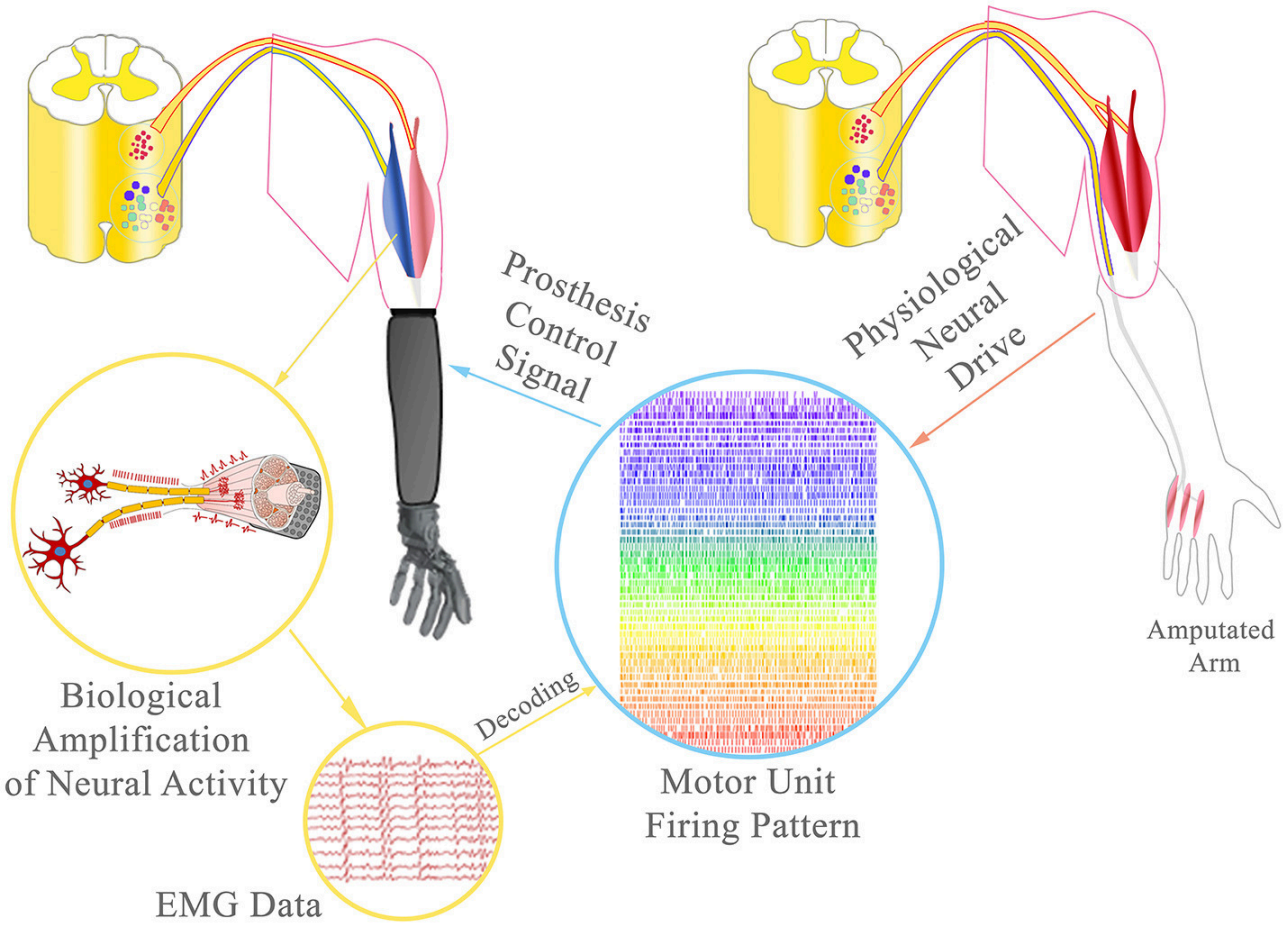
Broadband prosthetic interface: During TMR, the amputated ulnar nerve was rerouted to the short/medial head of the biceps to provide additional EMG signals. Using multichannel EMG electrodes, single motor unit activity can be decoded from the EMG data and the neural drive of the ulnar nerve to the intrisic hand musculature estimated. This information could allow the delivery of extremely precise control signals, ultimately with the same accuracy as physiologically reached.

Despite the promising clinical translation of electrodes implanted in muscles, the information extracted from the recorded EMG signals is still associated to the activation of large portions of muscle tissue. For example, simulation studies have shown that the detection radius of the IMES electrodes may reach 8 mm (Lowery et al., 2006). More selective signals may be obtained by reducing the electrode active area. The ultimate limit of information extraction from EMG signals is the quantum of the electrophysiological muscle activation, i.e., the motor unit action potential (Figure 2). The associated neural information is that of a single efferent nerve fiber. Decoding EMG signals at the level of motor units would provide direct access to the full neural information of the innervating nerve motor fibers. Decoding EMG signals at this fine scale requires more selective detection sites and, at the same time, greater density of electrodes (spatial sampling). The principle of spatial sampling with small individual electrodes has been extensively applied for surface EMG systems (Hahne et al., 2012; Muceli and Farina, 2012; Ison et al., 2015) and currently this technology allows the decoding of the neural drive to muscles by blind source separation methods (Farina and Holobar, 2016). Recently, as a proof of concept, these systems have been used to decode the neural activation of motor nerve fibers following TMR and the motor neuron behavior has been mapped into control signals for prostheses (Kapelner et al., 2015; Farina et al., 2017). It was shown that this approach at the motor unit level is theoretically superior to classic pattern recognition of the interference EMG using global parameters in TMR patients (Farina et al., 2017). As we discussed previously (Farina et al., 2017), the proposed approach, that has been proven with non-invasive high-density EMG electrode grids, could be translated to implanted grids. The advantage of implanted systems would be the possibility of recording from a greater portion of the muscle, and the increased robustness and decreased variability of the recordings with respect to skin mounting. For example, epimysial implantation of electrode grids would provide EMG signals that do not depend on the patient's subcutaneous tissues and that do not shift in location over repetitive use of the prosthesis.

Recently, high-density spatial sampling of EMG signals has been implemented in intramuscular electrodes (Farina et al., 2008; Muceli et al., 2015). These systems, suitable for acute implants (a few hours to a few days, with percutaneous wires), allow the identification of the activity of multiple motor neurons, identified with similar blind source separation techniques as developed for high-density non-invasive electrode grids (Muceli et al., 2015; Negro et al., 2016). However, for clinical translation, multichannel EMG electrodes should be chronically implanted. Moreover, the decoding into individual motor units should be implemented with algorithms running in real-time and embedded in wearable electronics. Intramuscular signals have a broader bandwidth than the surface signals and are therefore sampled at higher frequencies. Online decomposition of multichannel intramuscular signals is not possible yet. However, we have recently proposed a method for real-time decomposition of single-channel intramuscular EMG (Karimimehr et al., 2017) that can be extended to multiple channels. Ultimately, chronically implanted multichannel EMG systems should be decoded online to provide the full neural information of the motor nerve fibers. This achievement would in principle establish a control interface for as many functions as the human hand or upper extremity can naturally perform.

## Decomposition of multichannel EMG signals

The availability of several recordings of muscle fiber electrical activities theoretically allows the separation of the sources (discharge timings of motor neurons) from the convolutive mixing matrix. This can be performed by blind source separation methods that exploit, e.g., the sparseness property of the sources (Farina and Holobar, 2016). An assumption of these methods is that the number of observations is greater than the number of sources and this imposes a high spatial sampling, as discussed above. Blind separation of EMG signals has been demonstrated and validated in the past decade (Holobar and Farina, 2014) and has been tested on both invasive and non-invasive muscle recordings (Negro et al., 2016). Nonetheless, the conditions in which these methods have been applied are mainly constrained laboratory tests, during muscle contractions at constant or slow-varying force and in isometric conditions (Farina and Holobar, 2016). The extension of these methods to more general conditions is challenging because of the strong non-stationarity of the sources, artifacts, and brief activation intervals. These problems are further exacerbated by the need for online separation, which imposes constraints on the amount of data available for each processing intervals.

## Combination of nerve transfers, implantable multichannel EMG systems, and EMG decomposition

Recent neuro-histological analyses of peripheral nerves indicate that an average of approximately 25,000 efferent nerve fibers control the upper extremity function and thereof only approximately 1,800 motor nerve fibers ultimately control intrinsic hand function (Gesslbauer et al., in review). Re-routing these fibers to reinnervate residual stump muscles re-establishes motor unit function (Bergmeister K. et al., 2016). The hyper-reinnervation of a single muscle by a multifascicular nerve with a greater fiber input will lead to a higher number of functional motor units in the targeted muscle with respect to its natural innervation (Kuiken et al., 1995; Kapelner et al., 2016). Additionally, the nerve's individual fascicles may innervate certain compartments of the muscle that could be controlled independently from each other. Thus, a single muscle may serve as an amplifier (bio-screen) to the many fibers of a multi-fascicular nerve (Kapelner et al., 2015). By recording these targeted muscles with implantable multichannel EMG technology (Farina et al., 2008; Muceli et al., 2015), it would be possible to identify the clusters of muscle fibers that are innervated by each nerve fascicle and identify the behavior of motor neurons for each fascicle. This decoding approach is feasible, as shown in laboratory acute conditions in healthy (Muceli et al., 2015; Negro et al., 2016) as well as TMR patients (Farina et al., 2017). Considering the relatively small number of motor nerve fibers innervating the intrinsic musculature of the hand (only ~1,800), it is in principle possible to decode the full neural drive to the intrisic hand musculature with high resolution multichannel electrode systems. Thereby, the neural drive of a multi-fascicular (multi-modal) nerve could be identified in a single targeted muscle and the entire neural drive to the hand musculature could be decoded from several targeted muscles (Figure 2).

For example, in a transhumeral amputee, the redundant heads of the biceps and triceps muscles have a sufficient number of muscle fibers to receive the full innervation of the median and ulnar nerve, according to recent nerve fiber counting (Gesslbauer et al., in review). In this case, the multi-fascicular (multi-modal) nerves controlling intrinsic hand function would be represented as fascicular territories into the compartments of the two targeted muscles. By implanting multichannel EMG electrodes (either epimysially or intramuscularly) in these muscles, it would be possible to decode the activity of the separate populations of motor neurons for each fascicular territory. This information would allow the delivery of extremely precise control signals, ultimately with the same accuracy as physiologically reached.

## Challenges and conclusions

We illustrated the concept of an intuitive broadband myoelectric interface to improve prosthetic control. The proposed approach of decoding efferent nerve activity for control with a combination of surgical interventions, implanted electrode technology, and multichannel signal processing is supported by continuous advances in all these areas. Nonetheless, several challenges remain for the translation of these advances into clinical prosthetic systems. The main difficulties relate to the integration of tens to hundreds of recording sites in implanted EMG sensors and the wireless transmission of the signals on a large bandwidth, with high signal-to-noise ratio and with limited artifacts. Moreover, the recordings should be powered and stable over time for several years. Another set of limitations is related to the online robust processing of the EMG for extracting the constituent sources, in non-stationary conditions, during brief contractions, and with limited processing delay (within few hundreds ms). Once decoded, the sources need to be automatically associated to DoFs which is also a challenge. Despite these challenges, we believe the proposed scheme is more promising than current alternative research pathways. For example, an alternative approach is the direct recording from efferent fibers in peripheral nerves (Micera et al., 2011; Carboni et al., 2016; Ng et al., 2016), which however presents problems related to low signal amplitude and signal-to-noise ratio, small number of identified spike patterns, and potential intraneural damage (Navarro et al., 2005; Carboni et al., 2016). In comparison, the presented strategy may provide a safer and more robust method to operate modern prostheses with functions closer to the biological ones.

## Author contributions

KB, IV, SM, OA, DF designed the concept; all authors contributed their specific expertise, wrote the manuscript, and revised it critically.

### Conflict of interest statement

The authors declare that the research was conducted in the absence of any commercial or financial relationships that could be construed as a potential conflict of interest.
